# Use of nicotine products and tobacco cessation in Swiss primary care: Cross-sectional data from the Sentinella network

**DOI:** 10.1016/j.pmedr.2025.103013

**Published:** 2025-02-17

**Authors:** Marc Huguenot, Christina Hempel-Bruder, Ines Habfast-Robertson, Eva Guettinger, Isabelle Jacot-Sadowski, Julian Jakob, Reto Auer, Kevin Selby

**Affiliations:** a Department of Internal Medicine, Aarberg Hospital, Aarberg, Switzerland; b University Center for Primary Care and Public Health (Unisanté), University of Lausanne, Switzerland; c Institute of Primary Health Care (BIHAM), University of Bern, Switzerland; d Department of Paediatrics, University Hospital Bern, Inselspital, Bern, Switzerland; e Graduate School for Health Sciences, University of Bern, Switzerland

**Keywords:** Primary care, Tobacco, Nicotine products, Smoking cessation

## Abstract

**Introduction:**

We aimed to characterize current tobacco and nicotine product use and tobacco cessation efforts in Swiss primary care, including the prescription of medications and recommendation of vapes to quit smoking.

**Methods:**

Cross-sectional study from pediatricians and primary care physicians (PCPs) in the Swiss Sentinella network (practice-based network to monitor infectious diseases). PCPs collected data from 30 consecutive patients ≥12 years of age between September and December 2021. Patient data included age, gender, nicotine products use, plans to quit, and time discussing smoking cessation. PCP data were their use of medications, follow-up appointments, and vapes for quitting smoking.

**Results:**

Eighty-nine of 168 PCPs participated (53 %) and collected data on 2438 patients, of whom 523 (21,5 %) used a nicotine product within seven days, of whom 88 % smoked cigarettes. Among the 106 (20 %) who planned to quit smoking, 16 (15 %) planned to use nicotine replacement therapy, nine (9 %) varenicline, six (6 %) vapes, five (5 %) bupropion, and 57 no treatment (54 %). Moreover, 236 (46 %) of 523 patients using nicotine products received one to five minutes of cessation advice, 80 (16 %) six to ten minutes, and 17 (3 %) >10 min. Half of PCPs offered follow-up and medications to ≥50 % of patients planning to quit, while 52 % never recommended vapes.

**Conclusion:**

The use of nicotine products remains common among primary care patients, the majority of whom smoke cigarettes. Nicotine products without tobacco remain relatively rare. After the consultation, one in five patients using nicotine products planned to quit, the majority without any aid.

## Highlights

• One in five patients seen in primary care use nicotine products, mostly cigarettes.
• One in five patients using nicotine products plan to quit within the next three months.
• After seeing their primary care physician, over half plan to quit without help.
• Vapes are rarely used as quit aids, as most physicians perceive them as harmful.

## 1 Introduction

About a quarter of the Swiss population regularly uses tobacco, mainly in the form of conventional cigarettes, a proportion that has mostly remained stable since 2007 in women and 2017 in men (Addiction Suisse [Addictions Switzerland], 2024). Specifically, in the 2022 Swiss Health Survey, 18 % of the Swiss population aged 15 years and older reported smoking daily and 7 % to smoke occasionally, of whom 89 % smoked only cigarettes and 8 % cigarettes and another product (Addiction Suisse [Addictions Switzerland], 2024). Three percent of the adult population has used a vape within the last week. Vapes have been commercially available in Switzerland for about fifteen years, but the sale of nicotine-containing e-liquids was banned until 2018, when they became available up to 20 mg/ml. The situation again changed in October 2024 (after the current study) with the passage of a federal tobacco regulation act similar to the European tobacco regulations of 2014, with a nationwide minimum age of 18 years (Federal Office of Public Health, 2025). Vapes are not regulated as a pharmaceutical product in Switzerland and cannot thus technically be prescribed by physicians to their patients, as would be pharmacological nicotine replacement therapy (NRT). Physicians can recommend using vapes to their patients, who would then go buy them in a retail shop at their own costs and own risk. Heated tobacco products such as IQOS®, Ploom® or Glo® became available in 2015.Tobacco smoking remains the leading cause of preventable morbidity and mortality in Switzerland. The use of vapes, also called electronic cigarettes, and other nicotine products is less well described, and there are no recent data on their reported use in patients of primary care physicians (PCPs). Furthermore, the prevalence of heated tobacco products, shisha and snus among primary care patients in Switzerland is not known.

PCPs play an essential role in delivering cost-saving interventions to reduce the burden of tobacco in the population (Bassong et al., 2020; Stead et al., 2013). There is clear evidence that medications like varenicline, buproprion, and combination NRT can double the chances of quitting smoking (Cahill et al., 2013). Swiss recommendations for prevention in primary care strongly advocate screening for tobacco use and delivering proven interventions (Unisante, 2024). Internationally, tobacco cessation medications are underutilized, particularly in comparison with the management of other cardiovascular risk factors (Bernstein et al., 2013). In Switzerland, 60 % of smokers expect their doctor to talk about smoking during consultations and 51 % want advice on how to quit (Cornuz et al., 2018). When discussions with daily smokers were examined in the Sentinella network in 1996, 32 % of discussions resulted in a prescription or recommendation of some sort of quit aid, though the type of aid was missing in most cases (translation of relevant section in the Supplement) (Egli and Cloetta, 1998). In 2008, a series of Swiss patients who smoke were asked after a consultation if they discussed quitting; 10 % reported such a discussion and 9 % received help, despite 81 % being queried about smoking (von Garnier et al., 2008). More recent data are not available.

Recommendations have also become more complex with the advent of vapes and there are no recent data available to inform decision makers. Swiss PCPs receive conflicting information about how to answer questions about new nicotine products. On the one hand, there are concerns regarding the safety of vapes considering reports of lung injury in the United States and some evidence that vaping could be associated with smoking initiation (Al-Hamdani and Manly, 2021). On the other hand, they are considered less harmful and effective for tobacco cessation compared to traditional NRTs according to recent randomized trials (Lindson et al., 2024). Little is known about how Swiss PCPs are advising their patients in the context of widely divergent recommendations from different experts. This information is critical as the Swiss Medical Association update their training program, Evidence-Based Prevention in Primary Care, and the preventive clinical guidelines accordingly. Cost is an important barrier in Switzerland because many patients in primary care have high annual deductibles, NRT are expensive and not reimbursed by insurance (Jakob et al., 2022), there is no available generic version of varenicline, and cytisine is not autorized by the Swiss agency for therapeutic products (Swissmedic).

We analyzed the current prevalence of nicotine use (with and without tobacco) and status of tobacco cessation efforts in general practice, with a focus on time spent discussing tobacco cessation and the prescription of pharmacologic quit aids and vapes. The data collected will help to improve the training of PCPs in the management of their patients who smoke tobacco.

## 2 Methods

### 2.1 Study setting and data collection

This is a cross-sectional study of patients who were consulting their PCP or pediatrician in the national Sentinella network. The Swiss Sentinella Surveillance network was established in 1986 and at the time of this study had 168 PCPs and pediatricians who work in private practices (Federal Office of Public Health, 2024; Excoffier et al., 2018; Braun et al., 2019). The network collects epidemiological data for the surveillance of common communicable diseases and other pathologies in PCPs practice. It is a joint project conducted with practicing general practitioners, pediatricians, the Federal Office of Public Health, and the seven Swiss academic institutes for family medicine.

PCPs and pediatricians from the network, who we refer to collectively as PCPs for simplification, were recruited to collect data from 30 consecutive patients aged at least 12 years old, seen during non-urgent consultations, and complete a questionnaire about their practices related to tobacco cessation. Data were collected between September and December 2021, a period with relatively few COVID-19 restrictions and after the initial roll-out of COVID-19 vaccines in Switzerland. It is the PCPs who briefly noted the characteristics of patients and the consultations; patients did not themselves complete questionnaires. PCPs could choose between two options for including patients: a) consecutively include 30 patients seen in consultation (over the number of half-days required); b) consecutively include the first two patients per half-day. They had to include all patients in the same way (a or b). In addition, PCPs had the option of completing the questionnaire online or on paper. The second data source was an electronic questionnaire completed by physicians to collect information about their self-reported practices in general for tobacco cessation and opinions regarding the use of vapes for tobacco cessation. The ethics committee of the Canton of Bern waived approval for this study because all patient data were irreversibly anonymized, and PCPs were only providing their professional opinion.

### 2.2 Measures and outcomes

For patient information, we developed a data collection form in French and German based on previous studies (English translation in the supplement) (Braun et al., 2019) that PCPs could complete in two to three minutes directly after consultations. To facilitate and standardize data collection, we gave PCP's an algorithm showing the procedure to follow (supplement), explanations of the data we asked for and examples of completed data collection forms. Data collected for each patient included age, gender, and whether the patient had used a nicotine product within the last seven days. For patients answering yes they had used a nicotine product, further information was collected through PCP's about which nicotine product(s) they used regularly, their previous quit attempt(s), and whether they had used a quit aid. Nicotine products were categorized as those with tobacco (ex: cigarettes, pipes, heated tobacco, etc.) and those without (ex: vapes, nicotine pouches, nicotine replacement therapies, etc.) Cannabis was included in the category with tobacco because >80 % of cannabis users smoke joints with tobacco (Baggio et al., 2014). Patients were then asked about their intentions to quit or reduce their consumption within the next three months. Finally, PCPs provided information about the time spent for the overall consultation and time spent for smoking cessation.

The questionnaire completed by PCPs was about their approach to smoking cessation (full questionnaire in Supplement); when they prescribe medicines and suggest follow-up to help quit; and the average length (in minutes) of a stop smoking/stop nicotine product consumption discussion during a non-emergency consultation (supplement). We also asked their opinion concerning the harmfulness of different nicotine containing products on a scale of zero to 10 (10 being most harmful). The Swiss Sentinella Surveillance network provided additional information about most PCPs including the geographical region of Switzerland where their practice is located (Zurich, Lake Geneva region, Central Switzerland, Central Plateau, Northwest, Ticino, Eastern region), and the local density (rural, intermediate or urban).

### 2.3 Statistical analyses

We performed descriptive statistics to present the sample of patients and the PCPs who contributed data. Specifically, we calculated the proportion of patients who had used one or more nicotine product in the last seven days, divided into those with and without tobacco, accounting for dual or multiple users. The remainder of patient analyses focused on those who consumed nicotine products. For the prescription of quit aids, we focused on those planning to quit or reduce their consumption. Given our focus on describing current practices, we did not perform multivariable regression. All analyses were performed with STATA version 18 (StataCorp LP, College Station, TX, USA) and Microsoft Excel.

## 3 Results

Of the 168 PCPs in the Sentinella network invited to participate, 89 collected data from their patients and completed the electronic questionnaire (53 %). PCPs collected data from 2438 of their patients (1133 (46 %) men and 1305 women (54 %)), of whom 523 (21 %) used a nicotine product within the last 7 days (Table 1). Use of nicotine products with and without tobacco peaked at 31 % of patients aged 19 to 39 years and was lowest at 7 % of those aged 12 to 19. Among those having used a nicotine product, 271 (52 %) were men versus 252 (48 %) women; 237 (45 %) were aged 40–64 years, 141 (27 %) 65 years old or older, 130 (25 %) were aged 19–39 years, and 15 (3 %) were less than 19 years old (Table 1).

**Table 1:** Characteristics of consecutive patients seen in Swiss primary care between September and December 2021, stratified by those who reported being nicotine consumers and non-consumers (*n* = 2438).

	Users of nicotine products (with or without tobacco)	Non-consumers	Entire cohort
**Number of nicotine users**	523 (21 %)	1915 (79 %)	2438
**Gender**			
Women	252 (48 %)	1053 (55 %)	1305 (54 %)
Men	271 (52 %)	862 (45 %)	1133 (46 %)
**Age categories**			
< 19 years old	15 (3 %)	188 (10 %)	203 (8 %)
19–39 years old	130 (25 %)	288 (15 %)	418 (17 %)
40–64 years old	237 (45 %)	620 (32 %)	857 (35 %)
≥ 65 years old	141 (27 %)	819 (43 %)	960 (39 %)

**Fig. 1:**
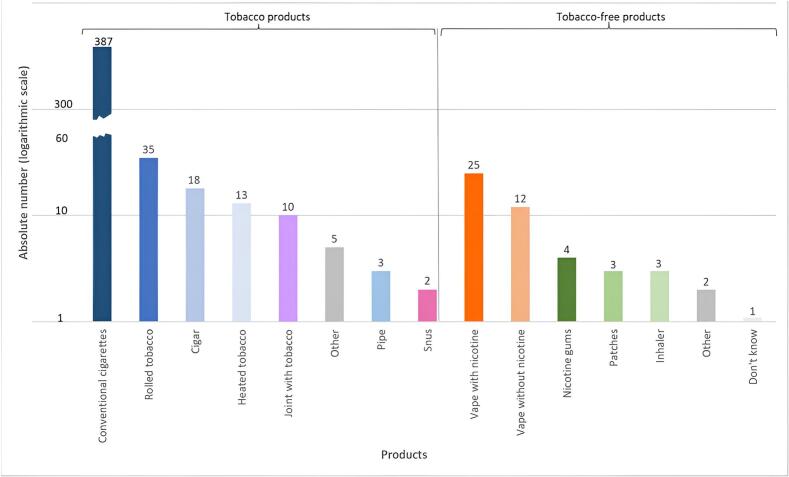
Nicotine products consumed by 523 patients seen in Swiss primary care between September and December 2021. Each patient could report using up to four products.

Of 523 nicotine users, the type of consummation was missing for 46 (9 %). Of the 487 remaining nicotine users, the vast majority, 473 (97 %), consumed tobacco products. Among them, 387 (82 %), consumed conventional cigarettes, 28 (7 %) rolled tobacco, 16 (4 %) cigars (Fig. 1). Interestingly, 49 (10 %) had a dual consumption with a second tobacco product. Most of them consumed cannabis (47 %) or rolled tobacco (22 %) as a second product. Among 409 people who primarily smoked cigarettes or rolled cigarettes, 179 (44 %) smoked 10 cigarettes per day or less, 167 (41 %) smoked between 10 and 20 cigarettes per day, 57 (14 %) smoked more than 20 per day, and 6 people (1.5 %) had missing data. 50 of the 487 nicotine users (10 %) used nicotine products without tobacco (Fig. 1). Among them, 25 (50 %) used vapes with nicotine, 12 (24 %) vapes without nicotine, and 10 (20 %) pharmacological nicotine substitutes. The majority (75 %) of those using products without tobacco were dual users (with and without tobacco). Among the 15 people aged 12 to 19 who had consumed a nicotine product, 13 (87 %) was with tobacco and two (13 %) was without.

Among the 523 patients using nicotine products, 37 % had never made a previous quit attempt of longer than 24 h. In the past, the majority (63 %) had not used a quit aid method, 31 % a nicotine replacement therapy (14 % gums, 13 % patches, 2 % spray or inhaler), 7 % medical advice, 6 % complementary medicine, 3 % a vape, and 2 % medication (bupropion, varenicline).

**Fig. 2:**
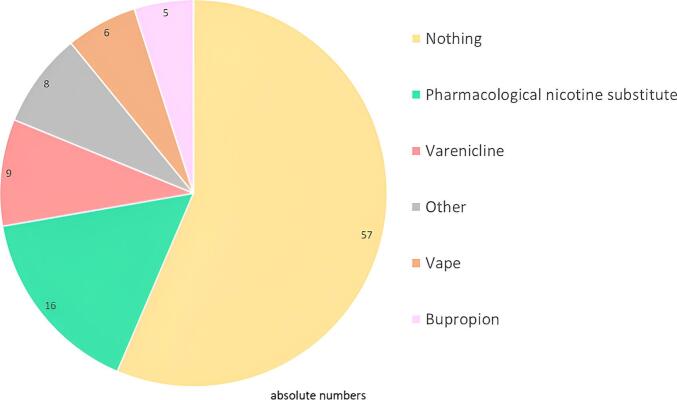
Tobacco cessation aids prescribed or recommended by Swiss primary care physicians to patients seen between September and December 2021 who were planning to quit or reduce their consumption over the next three months (*n* = 106).

Of 523 patients using nicotine products, 106 (20 %) planned to quit within 3 months, 43 (8 %) planned to reduce their consumption, and 307 (59 %) planned no change. The proportion who planned to quit was similar between different groups based on level of consumption and different types of products. Among those who planned to quit or reduce their consummation in the next three months, the majority (54 %) planned to not use a cessation aid after discussion with their PCP (Fig. 2). Among the remaining patients, 15 % planned for NRT, 9 % varenicline, 6 % vaping, 5 % bupropion, and 8 % something else. Among those who planned to reduce their consuming, 49 % planned to have no help, 14 % NRT or varenicline or something else and 5 % vaping. Interestingly, among those who had no intention to quit, 2 % still planned to use a pharmacological nicotine substitute or varenicline.

**Fig. 3:**
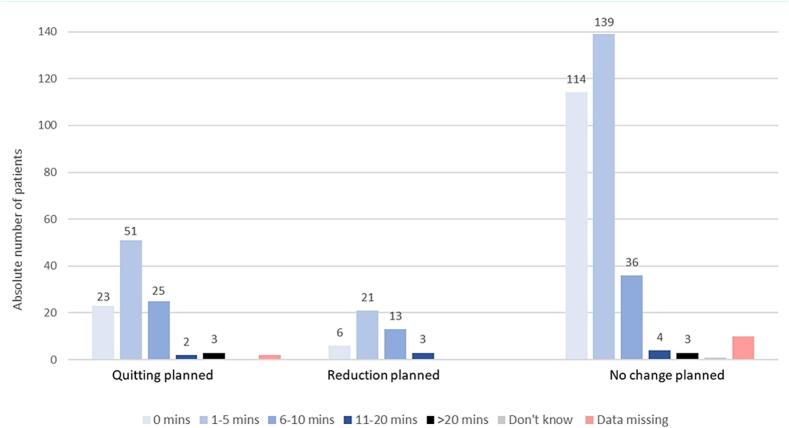
Time spent by Swiss primary care physicians discussing tobacco cessation with individual, consecutive patients between September and December 2021, stratified by the patient's intentions to quit or not during the next three month (n = 2438 patients).

Moreover, among the 523 consumers, 474 (91 %) patients received less than 10 min of cessation advice during their consultation with their PCP (Fig. 3). In those who considered to quit in the next three months, 99/106 (93 %) had 10 min or less of cessation advice. Among those who planned to reduce their consummation, 93 % (40/43) had 10 min or less of cessation advice. On those who had no plan to change their consumption, 94 % spent <10 min, with 139/307 (45 %) receiving 1–5 min of smoking cessation advice, 36/307 (12 %) 6–10 min, and another 114/307 (37 %) no advice at all (Fig. 3).

### 3.1 Results from primary care physician questionnaire

Characteristics of the 89 PCPs are shown in Table 2. Nine of the PCPs smoked and none of them vaped. There was a majority of men (62 %) aged between 45 and 65 years of age who were PCPs (85 % PCPs versus 13 % pediatricians). The frequency with which they self-reported offering different forms of help for quitting smoking is shown in Table 3. We found that about half of PCPs report offering close follow-up and medication to ≥50 % of patients planning a quit attempt. Answers were heterogeneous for the offer of ‘other help’, with the examples of acupuncture and hypnosis provided on the questionnaire. Finally, 52 % of PCPs never recommended using vapes to help quit, a proportion that was higher 40/65 (62 %) in PCPs in German-speaking cantons, and lower 6/23 (26 %) in PCPs in French and Italian-speaking cantons.

**Table 2:** Characteristics of Swiss primary care physicians participating between September and December 2021 and their region of practice (*n* = 89).

Characteristics	Number	Percent
**Gender**		
Women	33	38 %
Men	56	62 %
**Age category**		
20–45 years	19	21 %
45–65 years	56	63 %
≥65 years	12	13 %
**Used a nicotine product in the last 7 days**		
Yes	9	10 %
No or did not answer	80	90 %
**Regions of Switzerland (with cantons)**		
Lake Geneva (Geneva, Neuchatel, Vaud, Valais)	18	20 %
Mitelland (Bern, Fribourg, Jura)	15	17 %
Northwest (Aargau, Basel, Solothurn)	17	19 %
Central (Lucerne, Nidwalden, Obwalden, Schwyz, Uri, Zug)	7	8 %
Eastern and Zurich (Appenzell, Glarus, St Gallen, Schaffhausen, Thurgau, Zurich)	20	22 %
Ticino and Graubünden	11	12 %
**Specialty**		
General practitioner	76	85 %
Pediatrician	12	13 %
**Area of practice**		
Urban	72	81 %
Intermediary	8	9 %
Rural	8	9 %

**Table 3:** Self-reported practices for tobacco cessation and opinions regarding common nicotine products of 89 Swiss primary care physicians answering between September and December 2021. These practices and opinions are in general and not necessarily reflected during the consultations of the collection period.

Frequency with which different aids are offered	≥80 %	50–80 %	10–50 %	<10 %	never	I don't know / missing
Close follow-up as support for a quit attempt	24 (27 %)	25 (28 %)	18 (20 %)	12 (13 %)	4 (4 %)	6 (7 %)
Medication (NRT, bupropion, etc.)	19 (21 %)	22 (25 %)	23 (26 %)	14 (16 %)	7 (8 %)	4 (4 %)
Vape to help quit	2 (2 %)	6 (7 %)	7 (8 %)	21 (24 %)	46 (52 %)	7 (8 %)
Other help (acupuncture, hypnosis, etc.)	11 (12 %)	15 (17 %)	13 (15 %)	25 (28 %)	17 (19 %)	8 (9 %)
**Average length of consultations, depending on type**	**< 5** min	**5–10 min**	**11–20 min**	**21–30 min**	**> 30 min**	**I don't know / missing**
Brief intervention (non-motivated patient)	40 (45 %)	29 (33 %)	6 (7 %)	4 (4 %)	1 (1 %)	9 (10 %)
Motivational interviewing (ambivalent patient)	4 (4 %)	27 (30 %)	34 (38 %)	9 (10 %)	5 (6 %)	10 (11 %)
Intensive intervention (motivated patient)	2 (2 %)	23 (26 %)	29 (33 %)	18 (20 %)	3 (3 %)	14 (16 %)
**To what extent is this product harmful to its user?**	**Number responding “I don't know”**				**Average score (1–10 with 10 most harmful)**	
Cigarettes	3 (3 %)				8.8	
Heated tobacco products	12 (13 %)				7.2	
Vapes	5 (6 %)				6.5	
NRTs	5 (6 %)				3.7	
**To what extent is this product harmful to bystanders?**						
Cigarettes	2 (2 %)				7.8	
Heated tobacco products	23 (26 %)				6.1	
Vapes	25 (28 %)				5.4	
**To what extent is this product addictive?**						
Cigarettes	2 (2 %)				8.8	
Heated tobacco products	21 (24 %)				8.3	
Vapes	18 (20 %)				8.1	
NRTs	6 (7 %)				5.4	

NRT: Nicotine replacement therapies.

Finally, PCPs were asked about the perceived harmfulness of different products to its user and bystanders, as well their addictiveness (Table 3). About a quarter of respondents responded that they “didn't know” to what extent heated-tobacco products and vapes are harmful to bystanders and users. There was a gradient of harmfulness with cigarettes perceived as the most and NRTs as the least harmful, though the average score given was greater than five on 10 for vapes. The three products were seen as being similarly addictive.

## 4 Discussion

The use of nicotine products remains common among primary care patients, the majority of whom smoke cigarettes. Nicotine products without tobacco remain rare. Over one in five patients using nicotine products planned to quit in the next three months, the majority of whom discussed their plans less than 10 min with their PCP and did not plan to use a prescription quit aid. Most PCPs reported rarely or never recommending vapes for quitting, possibly linked to their high levels of perceived harmfulness of vapes.

According to the Swiss Health Survey, only 3 % of the population used a vape at least once a week, though that proportion rose to 25 % on the most recent lifestyle survey when asking 15-year-olds if they've vaped at least once in the last 30 days (Delgrande Jordan et al., 2023). Our data suggest that while spikes in vape use among teenagers have received considerable media attention, patients who vape regularly are rarely seen in primary care, even in the 12 to 19 years age group. Continuing medical education and other efforts to reinforce smoking cessation in primary care should continue to focus on quitting tobacco.

While survey data suggests that 59 % of people who smoke in Switzerland would like to quit at some point (Addiction Suisse [Addictions Switzerland], 2024), we measured 21 % who plan to quit in the next three months, who constitute 4 % of all patients over age 12 seen in primary care. In guidelines, these patients are the target group for intensive interventions including setting a quit date, the prescription of quit aids, and close follow-up (Cornuz et al., 2018). Physicians recorded that about half of these patients planned to use a quit aid and that most discussed less than 10 min with their PCP. The Sentinella network of PCPs also collected data in 1996 from 2194 daily smokers (not consecutive patients like our sampling). At that time, 31 % received 10 min, 33 % between 10 and 14 min, and 17 % 15 min of counseling, suggesting PCPs were able to take more time for discussions. However, only 32 % received some form of quit aid, suggesting the proportion receiving some sort of treatment has increased in the last 25 years. The decreased time available could be due to shortages in the number of PCPs and increasing number of comorbidities, limiting the time available to discuss a non-urgent issue like tobacco cessation (Golder et al., 2024). Swiss PCPs are also under increased pressure from health insurance companies to shorten their consultations. Now that Swiss PCPs can prescribe follow-up with trained psychologists and that nurse practitioners are expected to arrive in primary care, some more intensive discussions and follow-up could occur with different providers. The easy availability of pharmacists and vape shops could be other venues for tobacco cessation advice (Joss et al., 2021).

When we asked PCPs how often they suggest medication for quitting (Table 3), 46 % report offering medications ≥50 % of the time, while only 29 % of patients planning to quit intended to use a medication. This difference between what PCPs say they offer and plans after individual consultations could be due the fact that we collected data from single consultations while most patients have a longitudinal relationship with their PCP, patient refusal, or simply that PCPs overestimate what they actually do in practice. Previous qualitative studies have identified multiple barriers to the use of quit aids, from both PCPs and patients. PCPs frequently cite a lack of time, lack of effective treatments, and a lack of interest from patients (Zwar et al., 2006; Girvalaki et al., 2020). Many patients do not consider medications or help from their PCP as an important part of quitting, feeling that their success in quitting mostly depends on their personal motivation (Boesch et al., 2024).

In this sample, vapes were rarely used by patients in their previous quit attempts and in planned future quit attempts. This result is different from reports that vapes are now frequently used as quit aids, or that vapes are an effective means of quitting smoking (Lindson et al., 2024). One explanation is that our data were collected in 2021, before more recent results (Auer et al., 2024). There are also strong opposing positions between different groups of physicians who highlight the potential harmfulness of vapes rather than their potential as an attractive quit aid, a perception shared by many PCPs in our study. The geographical differences between western French speaking part and the Swiss-German speaking part reflect some of the polarization in the tobacco cessation community. The recent publication of the largest randomized controlled trial to date on vapes for smoking cessation, conducted in Switzerland, and the dissemination of results might alter this perception over time (Auer et al., 2024).

Strengths of this study include the collection of data from consecutive patients consulting for non-urgent reasons in private practices from throughout Switzerland. Data were collected about the same topic in this same network 25 years earlier. By asking about the use of any nicotine product in the last seven days, we could include patients who might not consider themselves as smokers. Multiple nicotine products could be recorded for each patient, to allow for collection about dual or multiple product users. Finally, because data are irreversibly anonymized before transfer to the Federal Office of Public Health, patients do not need to provide consent, which limits the number of patients refusing to participate.

Our study also had limitations. Notably, despite collecting data from 2438 consecutive primary care patients, the final numbers of patients using nicotine under 18 years of age or of dual or multiple users was quite small. While this reflects the low prevalence of these groups in primary care, we were not able to characterize their usage. Second, some patients, especially in younger age groups, may be reluctant to tell their physician they smoke, resulting in underreporting. Third, the use of nicotine products is evolving rapidly in Switzerland, meaning that these data from 2021 will need to be updated regularly. However, changes in usage are more pronounced in young adults, who are less often seen in primary care. Another limitation might be that PCP were more likely to talk longer than they normally do about nicotine use and smoking cessation because they knew data will be collected. Last, as the practitioners filled the questionnaire for the patients it could influence and there could be a response bias given the social desirability of helping patients quit smoking.

In conclusion, in this national sample of consecutive patients seen in Swiss primary care, the dominant nicotine product in use remains tobacco cigarettes. Vapes were rarely used, either in daily use or as quit aids, contrary to the widespread attention they have received in the media and evidence of their potential for harm reduction. Twenty-five years after a similar data collection, we found an increase in the use of quit aids and a worrying decrease in time spent discussing tobacco cessation. Given that tobacco remains the leading cause of preventable mortality in Switzerland, systematic change is needed to reinforce the help provided to current smokers in primary care. Basic insurance should provide access to smoking cessation counseling and nicotine replacement therapies without deductibles.

## Funding

This study did not receive any specific funding. Kevin Selby received salary support from the Leenaards Foundation. Reto Auer reports funding to test the efficacy, safety and toxicology of E-cigarettes by Swiss National Science Foundation (SNSF) [grant number IICT_33IC30_173552], Swiss Tobacco Prevention Fund (TPF) [grant number 19.017477], Swiss cancer research (SCR) [grant number KFS4744-02-2019] and Lunge Zürich.

## CRediT authorship contribution statement

**Marc Huguenot:** Writing – review & editing, Writing – original draft, Visualization, Formal analysis. **Christina Hempel-Bruder:** Writing – review & editing, Methodology, Investigation, Formal analysis, Conceptualization. **Ines Habfast-Robertson:** Writing – review & editing, Writing – original draft, Visualization, Formal analysis. **Eva Guettinger:** Writing – review & editing, Project administration, Conceptualization. **Isabelle Jacot-Sadowski:** Writing – review & editing, Conceptualization. **Julian Jakob:** Writing – review & editing, Project administration, Methodology, Conceptualization. **Reto Auer:** Writing – review & editing, Supervision, Project administration, Methodology, Investigation, Conceptualization. **Kevin Selby:** Writing – review & editing, Supervision, Project administration, Methodology, Investigation, Formal analysis, Data curation, Conceptualization.

## Declaration of competing interest

The authors declare that they have no known competing financial interests or personal relationships that could have appeared to influence the work reported in this paper.

## Data Availability

The authors do not have permission to share data.

## Appendix A Supplementary data

Supplementary material Image 1
