# Supplementary material for: Use of nicotine products and tobacco cessation in Swiss primary care: Cross-sectional data from the Sentinella network
Source: Prev Med Rep. 2025 Feb 17;51:103013. doi: 10.1016/j.pmedr.2025.103013 (PMC11908592; doi:10.1016/j.pmedr.2025.103013)
Supplement: Supplementary file 1 — Supplementary material [file mmc1.pdf]

NOTICE (NicOTIne use for smoking CEssation): a cross-sectional study of Swiss primary care physicians

Supplemental Materials

Sentinella findings regarding smoking cessation in 1996, translated……………………………………………………2

Data collection sheet NOTICE-PC 2021 (NicOTIne use for smoking CEssation in Primary Care)……………….3

Information sheet presenting the study to primary care physicians………………………………………………………..4

Questionnaire for primary care physicians………………………………………………………………………………………….9

Sentinella findings regarding smoking cessation in 1996, translated

English-language summary: Smoking is acknowledged as a serious public health problem by the medical profession and the public. In 1996, a survey was conducted to determine the frequency and the time spent for personal smoker counselling in primary care. The 20-59 age group was counselled most frequently, with a mean consulting time of 10-13 minutes. In more than 50% of such consultations, major illnesses were the reason for having the personal smoker counselling. Prescribing specific medication took place in only about 10% of these consultations.

Methods (from German): Registration criteria were age, gender of people who smoke cigarettes daily, time spent smoking advice in minutes, existence of a disease, initiative for smoking advice based on patient or doctor, if patient initiative: request for advice, prescription of medication or both. The result of the consultation regarding further advice was also collected, as well as the prescription of patch, spray or chewing gum and the recommendation or use of alternative methods.

Results (from German): Frequency of consultations: 220 doctors reported a total of 2,194 consultations in which smoking advice took place. This corresponded to an average of ten consultations per doctor. Based on the total number of doctor-patient contacts, the proportion of smoking consultations was 0.24% for general practitioners and internists, 0.01% for paediatricians. The spread of the reporting frequency per doctor was between 0 and 58. The proportion of general practitioners (11.2 cases) and internists (9.4 cases) was understandably much higher than that of paediatricians (0.5 cases). Patients of the age groups 30-39 and 40-49 were the most frequently counselled, the age group over 60 was represented far less frequently, and the least frequently patients under 20.

Time spent on average: the time required for general practitioners was 13.2 minutes, for internists 12.8 minutes and for paediatricians 10 minutes. The most frequent counselling times were 10 minutes (30.8%), 5 (18.9%) and 15 minutes (17.2%). Most consultations (715 / 32.8%) lasted between 10 and 14 minutes. Extent of demand for smoking advice In 2159 cases, the initiative for advice came from the patient in 36%, in 64% the doctor initiated the advice.

Existence of concomitant diseases: In 2080 cases, 24.3% had acute respiratory illnesses, in 26.3% an illness caused by smoking was an occasion for advice. Thus, 50.6% had significant illnesses that led to counselling. The remaining 49.4% was divided between patients with other illnesses or accidents (17.4%),on the other hand, on counselling without illness or accident (32.1%).

Frequency of prescriptions for specific medications: This question was only asked when the patient initiated smoking advice. The request for advice came in 37.6%, the request for a prescription only in 20.2% of the cases. The desire for both was 42.2% of the time.

Result of the consultation: Further consultations were planned in 40.7% of the cases. The prescription of a patch or spray was only carried out in 8.6%, the prescription of chewing gum in 1.3% of the cases. Alternative methods were provided in 2.3% of the cases, other, unspecified methods in 19.4% of the cases. In the possible combinations of methods, advice and additional prescription of patch or spray come first with 11.2%. All other combinations of methods were much rarer.

Data collection sheet NOTICE-PC 2021 (NicOTIne use for smoking CEssation in Primary Care)

Doctor ☐☐☐☐ Inclusion (option a or b) ☐ Week ☐☐ Sheet Nr. ☐

Pat.	1	2	3	4	5	6	7	8	9	10	11	12	13	14	15
Gender Mal (H), Fem (F), Trans* (T)															
Year of birth (≤ 2009) 19□□ or 20□□															
Consumption of nicotine products with or without tobacco in the last 7 days?	1= yes 2= no 3= discussion not possible 4= no data available														
	***STOP**: if not answer 1*														
Type(s) of product WITH tobacco (max 2 products/pers) and how many are consumed per day (number/day)	0= none 1= conventional cigarette 2= rolled tobacco 3= cigar 4= pipe 5= snus 6= heated tobacco (e.g. iQOS) 7= cannabis joint with tobacco 8= other 9= don't know 10= no data<br>number/day 0= 0 1= 1-5 2= 6-10 3= 11-15 4= 16-20 5= ≥ 21 6= don't know 7= no data														
Type(s) of product WITHOUT tobacco and daily consumption (number of doses/day or ml/week)	0= none 1= vape without nictoine 2= vape with nicotine 3= chewing gum 4= patches 5= spray 6= inhaler 7= nicotine pouch (snus without tobacco) 8= other 9= don't know 10= no data<br>doses/d or ml/week 0= 0 1=1- 5 2= 6-10 3=11- 19 4= ≥20 5= don't know 6= no data														
How many quit attempts of more than 24 hours in the past?	0= 0 1= 1 2= 2 3= 3 4= 4 5= ≥ 5 6= don't know 7= no data														
Quit aid method(s) used in t h e past (max 4 methods/ person)	0= none 1= gum 2= patches 3= spray 4= inhaler 5= bupropion 6= varenicline 7= vaporette 8= medical advice 9= complementary medicine														
Planning to stop consumption in the next 3 months?	0= No 1= want to stop 2= want to reduce 3= don't know 4= no data														
Quit-smoking method(s) prescribed or planned (two methods may be prescribed in parallel)	0= none 1= nicotine substitute 2= varenicline 3= bupropion 4= vaporette 5= other 6= don't know 7= no data														
Duration (in min) of consultation	0= 5-10 1= 11-20 2= 21-30 3= 31-45 4= >45 5= don't know 6= no data available														
Time (in min) spent on stop advice	0= 0 1= 1-5 2= 6-10 3= 11-20 4= 21-30 5= ≥30 6= don't know 7= no data														

NOTICE-PC (NicOTIne use for smoking Cessation in Primary Care) :
Data collection in the SENTINELLA network

Purpose of data collection

The aim of this study is to determine the prevalence of use of nicotine products with or without tobacco in general practice. We are also interested in the frequency of prescriptions for cessation aids and the time spent discussing smoking cessation during a general medical consultation. These data will provide important information for improving GP training as part of the *Vivre sans tabac* program (coordinated by the FMH), and will enable the development of new interventions to facilitate smoking cessation in general practice. Finally, we will compare our results with a SENTINELLA data collection on smoking cessation from 25 years ago, in 1996.

The aim of this study is therefore to measure the number of patients, aged ≥12 years, seen in general practice, who :

- have used a nicotine product with or without tobacco in the last 7 days ;
- smoke, vaporize or use other nicotine products ;
- have attempted to stop smoking in the past and with methods to help them stop;
- plan a quit attempt and, if so, with what help;

Procedure for filling in the "data collection" form

The form will be used over the coming weeks to collect data on the use of nicotine products with or without tobacco.

The aim is to complete the data collection form for **30 patients** according to the following reporting criteria:

- Ages 12 and up
- Non-urgent consultation lasting more than 5 minutes

To simplify filling in the form, you'll find an algorithm showing you the procedure to follow (**figure 1**) and detailed explanations of the questions (**table 1)** below.

When it comes to patient inclusion, you can choose between two options:

a. **Consecutive patients**
b. **The first two patients per half-day**

For option **a.** you include **30 patients consecutively** over the number of half-days required. For option **b.** you include the **first two patients** consecutively **per half-day**. In this way, you will need 15 half-days / 8 days for all 30 patients. Choose this option if you think data collection will take too much time per half-day.

Critère d'inclusion: Patient·e âgé·e ≥12 ans

Sexe et année de naissance<br><br>Consommation d'un produit nicotinique avec ou sans tabac au cours des 7 derniers jours

Non → STOP
Oui → Complétez le formulaire

Utilisation de produit <u>avec tabac</u> (max 2)<br>Non ↓<br>Oui → spécifiez le nombre par jour
Utilisation de produit <u>sans tabac</u> (max 2)<br>Non ↓<br>Oui → spécifiez la concentration de nicotine en mg/ml (vaporette) ou mg (remplacements nicotiniques)
Nombre de tentative(s) d'arrêt par le passé<br>Non ↓<br>Oui → spécifiez le nombre et les aides à l'arrêt utilisées (max 4)
Planification d'arrêt de consommation dans les 3 prochains mois<br>Non ↓<br>Oui → spécifiez les méthodes d'arrêt envisagées (max 2)
Durée totale (en min) de la consultation<br>Durée (en min) consacré aux conseils pour les produits à base de nicotine

Figure 1: The procedure for filling in

It is important that you include all patients in the same way (option **a** or **b**) and do not include them selectively. Notify your choice of inclusion method on the data collection sheet ("inclusion option a or b"). If a half-day is not suitable for collection (e.g., emergency or other irregular sequence), or if you forget to collect the first patients of the day, the missing half-days can be added at the end of the study. One page of the data collection sheet can accommodate 15 patients, so to complete the survey, two pages need to be filled in.

Table 1: Overview and explanation of data collection

Questions	Formulations proposed	Answers	Explanations
Gender		H = male F = female<br>T = trans*	Trans* includes transgender, transsexual, etc.
Year of birth		19___or 20___	STOP if <12 years<br>(i.e. born after 2009)
Use of nicotine products with or without tobacco over the past 7 days	Have you smoked or vaped in the last 7 days? Other tobacco products? Other nicotine products tobacco-free?	1= yes<br>2= no<br>3= no discussion possible<br>4= no data	Including the use of a nicotine-free vaporette.<br>Consumption even on one occasion.<br>STOP if no
Type(s) of products WITH tobacco (max 2 products/pers)	What tobacco products have you used? Have you smoked cannabis joints containing tobacco?	0= none<br>1= conventional cigarette 2= rolled tobacco<br>3= cigar<br>4= pipe<br>5= snus<br>6= Heated tobacco (e.g. iQOS)<br>7= cannabis joint with tobacco<br>8= other<br>9= don't know	The 2 most frequently used products if >2
How much is consumed per day (number/day)	How many cigarettes do you smoke a day? One pack = 20 cigarettes.<br>How many tobacco-containing products do you consume per day?	0 = 0<br>1= 1-5<br>2= 6-10<br>3= 11-15<br>4= 15-20<br>5= ≥ 21<br>6= don't know<br>7= no data	On average.<br>0 if smoked but does not smoke this product most days
Product type(s) Tobacco-free	Do you use a vaporette (with or without nicotine)?<br>Do you use nicotine replacement products?	0= none<br>1= nicotine-free vaporette<br>2= vaporette with nicotine<br>3= chewing gums 4= patches<br>5= spray<br>6=nicotine pouch (tobacco-free snus)<br>7= other	The 2 most frequently used products if >2.<br>Answers 3 to 5 concern nicotine replacement products (e.g. Nicorette).

		8= don't know<br>9= no data	
Daily consumption (number of doses/day or ml/week)	How many nicotine replacement doses per day? How many milliliters of liquid per week for your vaporette? A bottle of liquid is 10 ml.	0= 0<br>1= 1- 5<br>2= 6-10<br>3= 11- 19<br>4= ≥20<br>5= don't know<br>6= no data	If combining nicotine replacements, count 1 dose for patch + short-term replacements.
How many previous attempt(s) to stop for more than 24 hours?	Have you tried to quit in the past? How many times did it last at least a day?	0= 0<br>1= 1<br>2= 2<br>3= 3<br>4= 4<br>5= ≥ 5<br>6= don't know<br>7= no data	Stoppages >24 h. All quit attempts count since the start of consumption. An attempt to replace a tobacco-containing product (e.g. cigarettes) with a tobacco-free product (e.g. vaporette) is counted as an attempt to quit.<br>stop.
Method to help the stop used in the past (max 4 methods/patient)	When you have tried to quit, did you use a specific treatment or approach?	0= none<br>1= chewing gums 2= patches<br>3= spray<br>4= bupropion<br>5= varenicline<br>6= vaporette<br>7= Medical advice<br>8= Complementary medicine 9= Other<br>10= don't know<br>11= no data	Up to 4 stop-smoking methods/person. If >4 methods used, put the ones that seem to be the most effective.<br>important for the patient. Complementary medicine includes acupuncture, hypnosis, massage, etc. Chewing gums, patches and sprays are replacements nicotinics.
Planning to stop consumption in the next 3 months	Do you plan to try quitting in the next 3 months?	0= No<br>1= wants to stop<br>2= want to reduce 3= don't know<br>4= no data	If someone is planning to cut back in preparation for a stop, put "wants to stop".
Quit-smoking method(s) prescribed or planned (two methods may be prescribed in parallel)		0= none<br>1= nicotine substitute 2= varenicline<br>3= bupropion<br>4= vaporette<br>5= other<br>6= don't know<br>7= no data	Includes advice to use a nicotine substitute or vaporette without prescription. Other may include complementary medicine, physical activity, etc.

Duration (in min) of consultation		0= 5-10<br>1= 11-20<br>2= 21-30<br>3= 31-45<br>4= >45<br>5= don't know	Approximate duration, in minutes, of the complete consultation. We do not collect data for consultations <5 mins.
		6= no data	
Time (in min) spent on stop advice		0= 0<br>1= 1-5<br>2= 6-10<br>3= 11-20<br>4= 21-30<br>5= ≥30<br>6= don't know<br>7= no data	Approximate time, in minutes, devoted to talking about nicotine products with or without tobacco (quantifying consumption and talking about quitting).

We would like to highlight a few points:

1. Someone who has smoked a cigarette or used a nicotine product with or without tobacco in the last 7 days (even once) is considered a user. Conversely, a long-term smoker/vapotteur who has not used a nicotine product in the last 7 days is considered a non-user.
2. We make a distinction between the use of nicotine products with tobacco and without tobacco. Tobacco products include conventional cigarettes, rolled tobacco, cigars, pipes, snus, chewing tobacco, heated tobacco (e.g. iQOS) and cannabis joints containing tobacco. Each patient can specify up to 2 tobacco products consumed in the last 7 days.
3. Tobacco-free products include vaporettes (with and without nicotine), nicotine replacements (gum, tablets, spray, inhaler, patch), nicotine pouch (tobacco-free snus) and other similar products containing no tobacco.

Questionnaire on your practices and your perception of tobacco and/or nicotine products In addition to the patient data collection sheet, we have prepared an electronic questionnaire on the management of patients who use nicotine products with or without tobacco in your practice and your opinion about these products. Combined with the results of the prevalence survey, this will help us to improve training and develop needs-based interventions. We ask you to complete this questionnaire *only once* and *prior to data collection*. This survey is independent of the data collection and will be distributed to you separately.

Current problem
Around a quarter of the Swiss population regularly uses tobacco, mainly in the form of conventional cigarettes, and this proportion has hardly fallen in the last 10 years. Tobacco remains the leading cause of avoidable death in Switzerland. Despite clear evidence that pharmacotherapy can double the chances of quitting smoking, the use of medication for smoking cessation is under-utilized, particularly in comparison with the management of other cardiovascular risk factors. The use of vaporettes and other nicotine products is less well described, and there are no recent data on their use in family medicine.

Importance of the subject for general practice in Switzerland
General practitioners play an important role in promoting smoking cessation by offering treatment and support to their patients who smoke. In Switzerland, 60% of smokers expect their doctor to discuss smoking during consultations, and 51% want advice on how to quit. Recommendations have become more complex with the advent of vaporettes (i.e. electronic cigarettes), and there are no recent data available to inform decision-makers.

The data collected in this study will subsequently be used to improve GP training in the management of smoking patients and in the prescription of cessation aids, notably as part of the Vivre Sans Tabac program (coordinated by the FMH).

If you have any questions, please contact us by e-mail or telephone:

Unisanté - University Center for General Medicine

Dr. Med Kevin SelbyDr
Assistant doctorAssistant
Research Director
+41(0)31 631 58 70
kevin.selby@unisante.ch

. Med Christina Hempel-Bruder
doctor
christina.bruder@unisante.ch

In cooperation with

Prof. Dr. Med Reto Auer
Eva Güttinger
Julian Jakob

NOTICE EN

1 - What is your Sentinella doctor code?

---

2 - What's your gender?

◯ Men

◯ Woman

◯ Trans*

3 - What is your year of birth?

---

4 - Part 1: Your personal experiences

5 - Have you used nicotine products with or without tobacco in the last 7 days?

◯ Yes

◯ No

◯ I prefer not to answer

6 - If you answered no to the previous question, please specify your answer (multiple answers possible)

☐ I have never smoked cigarettes or other nicotine products, with or without tobacco (i.e. less than 100 times in my life).

☐ I've smoked more than 100 cigarettes in my life

☐ I've used a nicotine vaporette more than 100 times in my life.

☐ I have used other nicotine products with or without tobacco more than 100 times in my life (snus, shisha, nicotine substitutes, others).

7 - If you answered yes to the previous question, please specify your consumption (multiple answers possible)

☐ Conventional cigarettes

☐ Heated tobacco
☐ Snus
☐ Shisha
☐ Nicotine replacement
☐ Vaporette
☐ Other (specify below)

If other, please specify consumption:

---

8 - As a current (or former) smoker, what is (was) your average daily tobacco consumption?

◯ < 5 cigarettes/d
◯ ≥ 5 cigarettes/d < 10 cigarettes/d
◯ ≥ 10 cigarettes/d and < 1 pack/d
◯ ≥ 1 pack/d and < 2paquets/d
◯ ≥ 2 packs/d
◯ I don't know / not applicable

9 - As a vapoteu.se.r, do you consume liquid containing nicotine? If so, how much?

◯ No, I don't use nicotine
◯ Yes, < 5 mg/ml
◯ Yes, ≥ 5 - < 10 mg/ml
◯ Yes, ≥ 10 - < 18 mg/ml
◯ Yes, ≥ 18 mg/ml
◯ Don't know/not applicable

10 - As a current (or former) smoker, have you personally tried any of the following methods in an attempt to quit smoking? (multiple answers possible)

☐ Yes, nicotine replacement therapy
☐ Yes, varenicline
☐ Yes, bupropion
☐ Yes, vaporette
☐ Yes, snus
☐ Yes, heated tobacco
☐ Yes, hypnosis

☐ Yes, acupuncture

☐ Yes, other (specify below)

☐ No

☐ Don't know / not applicable If other (specify what):

---

11 - Have you ever attended a "Living without tobacco" continuing education course?

◯ Yes

◯ No

◯ Don't know / not applicable

12 - Would you like to participate in a "Living tobacco-free" continuing education course (either for the first time or as a refresher)?

◯ Yes, absolutely

◯ Rather yes

◯ Rather no

◯ Absolutely not

◯ Don't know / not applicable

13 - Have you taken any other "stop smoking" prevention courses?

◯ Yes (specify below)

◯ No

◯ Don't know / not applicable

If yes, please specify the training course(s) attended:

---

14 - Part 2: Your approach to smoking cessation with your patients

15 - In your practice, do you see patients under the age of 19?

◯ Yes

◯ No

---

16 - At what age do you first broach the subject of smoking with your patients?

◯ At any age (from childhood)
◯ With adolescents (≥ 12 years or similar)
◯ Only in adults (≥ 18 years)
◯ I don't know / not applicable

17 - Do you give advice to your patients aged < 19years to discourage smoking initiation?

◯ Yes
◯ No
◯ Don't know

18 - If you answered no to the previous question, please specify your reason(s)... (several answers possible)

☐ It could damage the therapeutic bond
☐ It's not effective
☐ It's not my role
☐ I don't feel comfortable talking about tobacco with my < 19 year old patients
☐ Other (specify below) If other (specify your answer):

---

19 - Do you give advice to your < 19-year-old patients to discourage initiation of vaporette?

◯ Yes
◯ No
◯ Don't know

20 - If you answered no to the previous question, please specify your reason(s) (multiple answers possible)

☐ It could damage the therapeutic bond

☐ It's not effective

☐ It's not my role

☐ I don't feel comfortable discussing vaping with my patients aged < 19

☐ Very few of my patients aged < 19 are confronted with vaporettes

☐ Other (specify below) If
other (specify):

---

21 - At a first consultation (a new case), what proportion of your patients, aged ≥ 12 years, do you ask if they are smokers (in the same way as a systematic measurement of vital constants)?

◯ ≥ 80% of cases

◯ 50 - < 80% of cases

◯ 10 - < 50% of cases

◯ < 10% of cases

◯ Never

◯ Don't know / not applicable

22 - If a patient is identified as a smoker, do you give advice on quitting smoking?

◯ Yes, in ≥ 80% of cases

◯ Yes, in 50 - < 80% of cases

◯ Yes, in 10 - < 50% of cases

◯ Yes, in < 10% of cases

◯ Never

◯ Don't know / not applicable

23 - If a patient is identified as a smoker, do you ask if he/she is ready to make a quit attempt?

◯ Yes, in ≥ 80% of cases

◯ Yes, in 50 - < 80% of cases

◯ Yes, in 10 - &alt; 50% of cases

◯ Yes, in < 10% of cases

◯ Never

◯ Don't know / not applicable

24 - In general, how often do you ask again whether your patients, aged ≥ 12 years, continue to smoke or have started smoking?

◯ At every consultation
◯ Several times a year
◯ Once a year
◯ Every 2 to 5 years
◯ I rarely or never ask if my patients smoke
◯ I don't know / not applicable

25 - With your patients who smoke, in what context(s) do you systematically raise the issue of smoking? (several answers possible)

☐ During an initial consultation, for whatever reason
☐ During a check-up (asymptomatic patient)
☐ When a chronic condition potentially related to smoking worsens (COPD, asthma, CV disease, etc.)
☐ suspicious findings on lung imaging (e.g. CT, X-ray)
☐ In the event of an emergency potentially related to smoking (e.g. bronchitis, URTI)
☐ At the patient's request
☐ Systematically, during almost all consultations
☐ I don't know / not applicable

26 - Do you talk about alternative products (shisha, vaporettes, heated tobacco or Snus)? (several answers possible)

☐ Yes, shisha
☐ Yes, vaporettes
☐ Yes, heated tobacco products
☐ Yes, Snus
☐ Yes, other (specify below)
☐ No
☐ Don't know / not applicable If
other, specify which product(s):

---

27 - Part 3: Prescription of smoking cessation medication and medical follow-up

28 - In your current practice, how often do you do you provide the following advice/treatment to patients who smoke?

	In ≥ 80% of cases	in 50 - < 80% of cases	In ≥ 10 - < 50% of cases	In < 10% of cases	Never	don't know / not applicable
A proposal for close follow-up for support during a quit attempt:	◯	◯	◯	◯	◯	◯
A medical prescription (nicotine substitutes, varenicline, bupropion):	◯	◯	◯	◯	◯	◯
The possibility of using the vaporette as a stop-smoking aid:	◯	◯	◯	◯	◯	◯
Other smoking cessation tips (acupuncture, hypnosis, ...):	◯	◯	◯	◯	◯	◯

29 - In general, after what period of time do you see or contact your patient again when he/she wishes to stop smoking?

◯ After a few days
◯ After ≤ 2 weeks
◯ After > 2 weeks to ≤ 4 weeks
◯ After > 4 weeks
◯ According to patient's desire.
◯ I don't set a follow-up right away and wait for him/her to contact me again
◯ I don't know / not applicable

30 - For each treatment/cessation aid below, please indicate how often you PROPOSE them (on a scale of 0: never to 3: always)

	0: never	1: rarely	2: often	3: always
Nicotine substitutes	◯	◯	◯	◯
Varenicline	◯	◯	◯	◯
Bupropion	◯	◯	◯	◯
Vaporette	◯	◯	◯	◯
Snus	◯	◯	◯	◯
Heated tobacco	◯	◯	◯	◯

**Hypnosis**	◯	◯	◯	◯
**Acupuncture**	◯	◯	◯	◯

31 - For each treatment/cessation aid below, please indicate how often you PRESCRIBE them (On a scale of 0: never to 3: always)

	0: never	1: rarely	2: often	3: always
**Nicotine substitutes**	◯	◯	◯	◯
**Varenicline**	◯	◯	◯	◯
**Bupropion**	◯	◯	◯	◯
**Vaporette**	◯	◯	◯	◯
**Snus**	◯	◯	◯	◯
**Heated tobacco**	◯	◯	◯	◯
**Hypnosis**	◯	◯	◯	◯
**Acupuncture**	◯	◯	◯	◯

32 - In your opinion, does nicotine contribute directly to the development of the following diseases (on a scale of 0-5)

	not at all	no	rather no	rather yes	yes	yes, absolutely	don't know
**Congenital malformations:**	◯	◯	◯	◯	◯	◯	◯
**Cardiovascular diseases:**	◯	◯	◯	◯	◯	◯	◯
**Cancers:**	◯	◯	◯	◯	◯	◯	◯
**COPD:**	◯	◯	◯	◯	◯	◯	◯

33 - On the subject of vaporettes, you:

◯ Systematically suggest it as an option to help stop smoking

◯ Offer it as a stop-smoking aid only to patients who do not want any other stop-smoking aid.

◯ Offer it as a stop-smoking option only to patients who report having failed to quit in the past with medication.

◯ Don't offer it as a stop-smoking option

◯ Don't recommend it to your patients

◯ Avoid any discussion about it

◯ Don't know / not applicable

34 - For what reason(s) do you suggest vaporettes as a cessation aid? (several answers possible)

☐ Based on current knowledge, it is very likely to be effective in smoking cessation

☐ It is certainly less harmful than conventional cigarettes

☐ Vaporettes are less expensive than nicotine substitutes

☐ Several of my patients have been able to quit smoking thanks to vaporettes.

☐ My patients prefer vaporettes to other cessation aids

☐ Other (specify below)

☐ Don't know / not applicable If other, please specify:

---

35 - When you suggest vaporette as a cessation aid option, what do you suggest as the nicotine concentration in e-liquids?

◯ I encourage starting with high nicotine concentrations

◯ I encourage using the lowest possible nicotine concentration

◯ I encourage the use of nicotine-free e-liquids

◯ I recommend discussing this with another person (specialists (at the vape shop), peers, etc).

◯ I don't know / not applicable

36 - What is your reason for not offering vaporettes as a cessation aid option? (several answers possible)

☐ We don't have enough data for me to feel comfortable advising it

☐ I'm afraid of acute pneumonitis, such as that seen in the United States.

☐ We already have other cessation aid options that have been proven effective

☐ Vaporettes are highly addictive and therefore do not solve the problem of nicotine dependence.

☐ I fear the use of vaporettes by teenagers.

☐ Other (specify below) If other, please specify:

---

37 - When your patient is interested in vaporettes, do you refer her/him to a vape-shop?

◯ Yes, I recommend a particular store that I know well

◯ Yes, but not in a particular store

◯ No, I recommend that the person find out for themselves

◯ No, I think it's up to the doctor to advise the patient (particularly on nicotine content and which model to have)

◯ No, because I don't recommend vaporettes

◯ I don't know / not applicable

38 - On the subject of "heated" tobacco products like IQOS, you :

◯ Systematically suggest them as an option when helping people to stop smoking.

◯ Offer them as a stop-smoking aid only to patients who do not want any other stop-smoking aid?

◯ Offer them as a stop-smoking aid only in patients who do not want medication, or vaporette, as a stop-smoking aid.

◯ Do not offer them as a stop-smoking aid.

◯ Advise your patients against them.

◯ Avoid any discussion about them

◯ Don't know / not applicable

39 - About SNUS, you :

◯ Systematically propose it as an option in smoking cessation assistance

◯ Propose it as a stop-smoking aid option only in patients who do not want other stop-smoking aid medication

◯ Offer it as a stop-smoking aid only in patients who do not want medication, or vaporette, as a stop-smoking aid.

◯ Do not offer it as a stop-smoking aid.

◯ Don't recommend it to your patients

◯ Avoid any discussion about it

◯ Don't know / not applicable

40 - Part 4: Perception of the risk and addictive nature of nicotine-containing vaporettes

41 - In your opinion, to what extent are the following nicotine-containing products harmful to the health OF THOSE WHO CONSUME THEM? (on a scale from 0 to 9) (0: no harm / 9: extremely harmful)

	1	2	3	4	5	6	7	8	9	I don't know

Cigarette:	○	○	○	○	○	○	○	○	○	○
A heated tobacco product (such as IQOS or Ploom):	○	○	○	○	○	○	○	○	○	○
The vaporette:	○	○	○	○	○	○	○	○	○	○
Nicotine replacement therapy (patches, gum, spray, etc):	○	○	○	○	○	○	○	○	○	○

42 - How harmful do you think the following items are TO THIRD PARTIES? (on a scale of 0 to 9) (0: no harm / 9: extremely harmful)

	1	2	3	4	5	6	7	8	9	Don't know
Passive smoke from cigarettes:	○	○	○	○	○	○	○	○	○	○
Passive emissions from heated tobacco products:	○	○	○	○	○	○	○	○	○	○
Passive vapor (aerosol) from vaporettes:	○	○	○	○	○	○	○	○	○	○

43 - In your opinion, how addictive are the following nicotine-containing products? (on a scale of 0 to 9) (0: not at all addictive / 9: extremely addictive)

	1	2	3	4	5	6	7	8	9	I don't know
Cigarette:	○	○	○	○	○	○	○	○	○	○
A heated tobacco product (such as IQOS or Ploom):	○	○	○	○	○	○	○	○	○	○
Vaporette (with nicotine):	○	○	○	○	○	○	○	○	○	○
Nicotine replacement therapy (patches, gum, spray, etc):	○	○	○	○	○	○	○	○	○	○

44 - Part 5: Organizational questions

45 - What is the average length of an anti-smoking discussion during a consultation at your practice?

	< 5 min	5 - 10 min	11 - 20 min	21 - 30 min	> 30 min	Don't know
During a brief intervention (unmotivated patient):	○	○	○	○	○	○
During a motivational interview (embivalent patient):	○	○	○	○	○	○

**During a sustained intervention (motivated patient):**	○	○	○	○	○	○

46 - Would you like to have a decision-support tool for the discussion around quitting smoking?

○ Yes, rather in paper format
○ Yes, rather in electronic format
○ No
○ Don't know / not applicable Remarks:

---

47 - Part 6: Political issues

48 - Numerous studies show that tobacco advertising influences consumption behavior. In Switzerland, tobacco advertising is prohibited only on television and radio. Some cantons have developed more ambitious measures. Would you support the following more ambitious measures regarding tobacco advertising at the federal level? (On a scale of 0 and 5)

	0: no, not at all	1: no	2: rather no	3: rather yes	4: yes	5: yes, absolutely	I don't know
**Ban tobacco advertising in the press:**	○	○	○	○	○	○	○
**Ban tobacco advertising on billboards:**	○	○	○	○	○	○	○
**Neutral packaging for tobacco products ("plain packaging"):**	○	○	○	○	○	○	○
**Ban tobacco advertising at point of sale:**	○	○	○	○	○	○	○
**Ban the visibility of tobacco products at point of sale:**	○	○	○	○	○	○	○
**Ban tobacco sponsorship:**	○	○	○	○	○	○	○
**Prohibit product placement:**	○	○	○	○	○	○	○

**Prohibit customer loyalty measures (e.g. customer cards, coupons, etc.):**	○	○	○	○	○	○	○
**Ban contests to win tobacco products:**	○	○	○	○	○	○	○
**Ban free samples:**	○	○	○	○	○	○	○

49 - Tobacco taxation is an effective prevention method. At present, the retail price of a pack of 20 cigarettes is between 6 and 9 francs, made up mainly of taxes (around 60%) and the manufacturers' and retailers' share (around 40%). (Tobacco tax 52.5%, VAT 7.2%, Fonds de prévention du tabagisme 0.3%, Fonds de cofinancement du tabac domestique 0.3% and the share of manufacturers and retailers. distributors 39.7%). Please indicate whether you agree or disagree with the following statements. (On a scale from 0 to 5)

	0: no, not at all	1: no	2: rather no	3: rather yes	4: yes	5: yes, absolutely	I don't know
**The sale of tobacco products entails more health and social costs than the money it generates for society through taxes:**	○	○	○	○	○	○	○
**Tobacco prices are set to rise:**	○	○	○	○	○	○	○
**Tobacco tax should be increased:**	○	○	○	○	○	○	○

50 - Tobacco tax revenue currently stands at CHF 4.50 per pack of 20 cigarettes, for a retail price of CHF 8.60. How many francs do you think the tobacco tax should be to offset the health and social costs of smoking?

○ 1-3 CHF (less than current tax)

○ 4-5 CHF (similar to the current tax at a selling price of 8.60 CHF)

○ 5-9 CHF

◯ 10-14 CHF

◯ 15-19 CHF

◯ ≥20 CHF

51 - Please indicate whether you agree or disagree with the following statements. (On a scale from 0 to 5)

	no, not at all	no	rather no	rather yes	yes	yes, absolutely	I don't know
**A tax should be levied on e-cigarettes:**	◯	◯	◯	◯	◯	◯	◯
**Advertising for e-cigarettes should be banned:**	◯	◯	◯	◯	◯	◯	◯
**E-cigarettes should be covered by health insurance as a smoking cessation treatment:**	◯	◯	◯	◯	◯	◯	◯
**A ban on issuing e-cigarettes to under-18s should be imposed:**	◯	◯	◯	◯	◯	◯	◯
